# Transthyretin Amyloidosis: Chaperone Concentration Changes and Increased Proteolysis in the Pathway to Disease

**DOI:** 10.1371/journal.pone.0125392

**Published:** 2015-07-06

**Authors:** Gonçalo da Costa, Cristina Ribeiro-Silva, Raquel Ribeiro, Samuel Gilberto, Ricardo A. Gomes, António Ferreira, Élia Mateus, Eduardo Barroso, Ana V. Coelho, Ana Ponces Freire, Carlos Cordeiro

**Affiliations:** 1 Centro de Química e Bioquímica, FCUL, Campo Grande, Lisboa, Portugal; 2 Instituto de Tecnologia Química e Biológica, Av. da República Estação Agronómica Nacional, Oeiras, Portugal; 3 Unidade de Transplantação, Hospital Curry Cabral, Lisboa, Portugal; Aligarh Muslim University, INDIA

## Abstract

Transthyretin amyloidosis is a conformational pathology characterized by the extracellular formation of amyloid deposits and the progressive impairment of the peripheral nervous system. Point mutations in this tetrameric plasma protein decrease its stability and are linked to disease onset and progression. Since non-mutated transthyretin also forms amyloid in systemic senile amyloidosis and some mutation bearers are asymptomatic throughout their lives, non-genetic factors must also be involved in transthyretin amyloidosis. We discovered, using a differential proteomics approach, that extracellular chaperones such as fibrinogen, clusterin, haptoglobin, alpha-1-anti-trypsin and 2-macroglobulin are overrepresented in transthyretin amyloidosis. Our data shows that a complex network of extracellular chaperones are over represented in human plasma and we speculate that they act synergistically to cope with amyloid prone proteins. Proteostasis may thus be as important as point mutations in transthyretin amyloidosis.

## Introduction

Transthyretin amyloidosis (ATTR) is an autosomal dominant degenerative disease characterized by the formation of amyloid fibril deposits, mainly composed of transthyretin (TTR), in different organs and tissues [1, 2]. These amyloid deposits hinder organ function, lead to their failure and, ultimately, death. ATTR has been associated, mainly by *in vitro* studies [3], with single amino acid substitutions in TTR, a plasma protein responsible for the transport of thyroxine and retinol in the blood, the latter via the association with the retinol-binding protein [4].

The only effective therapeutic option for ATTR is liver transplantation from cadaveric donors since plasma TTR is produced mainly in the liver. Moreover, domino liver transplant from ATTR patients, a practice recently introduced to obviate the shortage of livers available for transplantation, introduces TTR mutated forms in circulation, increasing the risk of ATTR development [5].

The main hypothesis for ATTR pathogenesis considers the tetramer instability favoring the dissociation to non-native monomeric species with the ability to self-associate. These soluble aggregates evolve to insoluble aggregates and amyloid fibers with the characteristic β-cross sheet structure found in several neurodegenerative disorders such as Alzheimer’s and Parkinson’s diseases [6].

This model, however, fails to explain two crucial aspects of amyloid formation. First, non-mutated TTR also forms amyloid, causing systemic senile amyloidosis [7]. Mutations only accelerate the intrinsic amyloidotic behavior of this protein. Second, time to disease onset varies by decades for different patients bearing the same mutation, and individuals transplanted with liver from transthyretin amyloidotic individuals present an amyloidotic behavior much faster that individual bearing amyloidogenic mutations [8]. Discordant disease progression in homozygote twins From Sweden and Spain was reported. In a case, one of the twins underwent liver transplantation, whereas the other is completely healthy, showing no symptoms 8 years after the onset of his brother disease [9, 10, 11]. It is also important to note that, homozygous ATTR V30M patients appear not to develop a more aggressive disease than heterozygous ones [12]. Genetic factors alone do not explain all the process for amyloid formation and other factors should be taken in consideration. These questions point to the involvement of multiple factors in ATTR development. Moreover, several studies described structural transient states [13,14,15] during fibrillation that under the correct circumstances, do not further convert into amyloid fibrils [16,17].

Proteome analysis in different biological samples is being increasingly used for clinical diagnosis and identification of protein biomarkers for the disease onset of various pathologies. 2-DE is still a promising research area for markers discovery [18]: the most important advantage of plasma proteomics is the prospect of a noninvasive and easy sampling system of diagnosis, which might reduce the need of any kind of biopsy. The practical utility of 2-DE for studies of the high abundance plasma proteome has been substantial. Because the first dimension of the procedure (isoelectric focusing) is exquisitely sensitive to molecular charge and the second dimension (SDS electrophoresis) is sensitive to polypeptide length, 2-DE is very effective at revealing genetic variants (about one-third of which differ in net charge from wild type (WT), proteolytic cleavages, and variations in sialic acid content [19].

In this work we evaluated the plasma proteome of ATTR individuals in relation to control individuals. We observed that several proteins were differentially expressed, namely extracellular chaperones, in agreement to what was previously observed in other amyloid diseases. Moreover we also observed increased proteolytic activity in ATTR plasma.

## Methods

### Plasma Collection

Blood samples from control individuals, ATTR patients (three females each, age range 26–33 years) and patients subjected to liver transplantation, both domino liver transplantation (DLT) and cadaveric donor liver transplantation (OLT) (four females each, age range 32–41 years) were collected by venous puncture to citrate containing tubes centrifuged at 1,800 *g* for 5 min at 4°C and the collected plasma was immediately frozen at -80°C until further analysis. All patients gave informed written consent and the protocol was approved according to EEC ethic rules and the Helsinki protocol. This study was approved by the Ethics committee of the Curry de Cabral Hospital.

#### Polyacrylamide Gel Electrophoresis.

Plasma proteins were separated by SDS-PAGE (12%), in mini-gel format (7x7 cm Tetra system from Bio-Rad). Twenty micrograms of plasma protein were loaded per lane. Protein concentration was determined by the Bradford protein assay, using bovine serum albumin (BSA) as a standard (Bio-Rad). Samples were diluted 10 fold in MilliQ water and mixed with reduction buffer (62.5 mM Tris-HCl, pH 6.8, 20% (v/v) Glycerol, 2% (w/v) SDS, 5% (v/v) -mercaptoetanol). Prior to electrophoresis, samples were heated at 100°C for 5 min. Protein bands were stained with Coomassie brilliant blue R-250.

### Two Dimensional Electrophoresis

Samples containing 350 μg of plasma protein were used for IEF (isoelectric focusing) using immobiline dry strips of 13 cm, respectively, with a non linear pH gradient from 3 to 11 [20, 21] (Amersham Biosciences). After IEF separation, proteins were reduced and alkylated in the dry strip. Second dimension SDS-PAGE was performed using 12% acrylamide/bysacrylamide, following general procedures. Protein spots were stained with Coomassie brilliant blue R-250.

### Image analysis

Gels images were acquired using the IMAGE SCANER (Amersham Biosciences) and the 2-DE gel images were exported to the Samespots Analysis software program (Nonlinear Dynamics). This software compares computer images of 2-DE gels to determine differential protein expression and accurately identifies increased or decreased proteins on the basis of spots staining intensity. When spots were detected, the original gel image was normalized by a filtration procedure and then the 3-D Gaussian spots were created. After spot detection in gels, a match set was generated and a standard gel image as a master map, made by merging the Gaussian images of each ATTR patients. This set was matched to the protein spots from healthy subjects primary gels. The protein spots in a comparison set of gels were quantitatively, qualitatively, and statistically analyzed.

### Western Blotting

For western blot analysis, proteins were transferred from the gel to PVDF membranes (Millipore) and stained with Ponceau S to monitor protein transfer. Membranes were blocked overnight at 4°C with TBS (10 mM Tris–HCl, 150 mM NaCl, pH 7.5) containing 5% skimmed milk. Afterwards, the membranes were incubated overnight at 4°C with the primary antibody used in TBS-T (TBS Buffer with 0.1% Tween 20) containing 1% skimmed milk. Antibodies used were: anti human TTR polyclonal antibody (DAKO) at a dilution of 1:5000, anti-fibrinogen (Calbiochem) at a dilution of 1:10000, e C-reactive protein (Santa Cruz Biotechnology) at a dilution of 1:500, Vitamin D-binding protein (DBP) (Santa Cruz Biotechnology) at a dilution of 1:500, Alpha-2-Macroglobulin (Santa Cruz Biotechnology) at a dilution of 1:500 and 1 anti-trypsin (Santa Cruz Biotechnology) at a dilution of 1:500.

Membranes were washed three times for 10 min each with TBS-T and incubated for 1 h at room temperature with anti-rabbit IgG (Roche) (at 1:10000 dilution) and anti-mouse IgG (Roche) (at 1:10000 dilution), for TTR and fibrinogen. Immunoreactivity was detected with Pierce ecl western blotting substrate, following the manufacturer’s instructions (Pierce). Each dried blot was scanned at 600 dpi (dots per inch) and saved as a TIFF file. Image analysis was performed using the public domain ImageJ program (National Institutes of Health, and available at http://rsb.info.nih.gov/ij/), using the “Gel Analysis” functions. Background correction was done using a “rolling ball” method with a radius of 4 times the width of a band. The result of the analysis is a value for each band which is proportional to the Integrated Density Value (IDV) of that band.

### In Gel Protein Digestion

Protein bands were manually excised from the gels and digested using trypsin as described [22, 23]. Briefly, gel protein bands were washed in milliQ water, distained in 50% acetonitrile (ACN and subsequently with 100% ACN. Cys residues were reduced with 10 mM DTT and alkylated with 50 mM iodoacetamide. Gel pieces were dried by centrifugation under vacuum and rehydrated in digestion buffer containing 50 mM NH_4_HCO_3_ and 6.7 ng/µL of trypsin (modified porcine trypsin, proteomics grade, Promega) at 4°C. After 30 min the supernatant was removed and discarded and 20 µL of 50 mM NH_4_HCO_3_ were added. Digestions were allowed to proceed at 37°C overnight (16–18 hours). After digestion, the remaining supernatant was removed and stored at -20°C.

### Mass Spectrometry

To identify target proteins, a MALDI-TOF/TOF 4800 plus mass spectrometer (Applied Biosystems, Foster City, CA, USA) was used, as previously described [24]. Desalting and concentration of tryptic peptides was carried out with home-made chromatographic microcolumns using GELoader tips packed with POROS R2 (Applied Biosystems, Foster City, CA, USA). The peptides were directly eluted from the micro columns onto the MALDI plate using α-ciano-4-hydroxycinnamic acid (5 mg.ml^-1^) in 50% (v/v) CH_3_CN with 0.1% (v/v) formic acid.

MS experiments were performed in positive reflectron mode for monoisotopic peptide mass determination. The mass spectrometer was externally calibrated using a mixture of des-Arg-Bradykinin (904.468 Da), angiotensin 1 (1296.685 Da), Glu-Fibrinopeptide B (1570.677 Da), ACTH (1–17) (2093.087 Da), and ACTH (18–39) (2465.199) (4700 Calibration Mix, Applied Biosystems, Foster City, CA, USA). MS spectra was collected in a result-independent acquisition mode, typically using 1000 laser shots per spectra and a fixed laser intensity of 3000 V. For tandem experiments, fifteen of the strongest precursors were selected for MS/MS, the weakest precursors being fragmented first. MS/MS analyses were performed using CID (Collision Induced Dissociation) with 1 kV collision energy and 1 x 10^6^ torr air pressure. 2000 laser shots were collected for each MS/MS spectrum using a fixed laser intensity of 4000V. Raw data were generated by the 4000 Series Explorer Software v3.0 RC1 (Applied Biosystems, Foster City, CA, USA) and tryptic peptide contaminant *m/z* peaks resulting from trypsin autodigestion (842.508 Da; 1045.564 Da; 2211.108 Da; 2225.119 Da) were excluded when generating the peptide mass list used for comparison with the theoretical tryptic digest. Proteins were identified by the GPS explorer (Applied Biosystem) and further confirmed using the ProteinPilot software (Applied Biosystem).

TTR quantification was performed as described [25, 26], briefly samples were desalted and concentrated using reverse phase

Poros R2 (Applied Biosystems) and eluted directly to the MALDI target AnchorChip (BrukerDaltonics, Bremen, Germany) with the appropriated matrix, according to the manufacturer procedure. Matrix solution of α-cyano-4-hydroxycinnamic acid (CHCA; Fluka) was prepared at a concentration of 10 μg/μL in 50% ACN with 0.1% TFA. Peptide mixtures were analyzed by MALDI-FTICR-MS in a Bruker Apex Ultra, Apollo II combi-source (Bruker Daltonics, Bremen, Germany), with a 7 Tesla magnet (Magnex corporation, Oxford UK). Monoisotopic peptide masses were determined using the SNAP 2 algorithm in Data Analysis software version 3.4 (BrukerDaltonics). External calibration was performed by using the BSA tryptic digest spectrum, processed and analyzed with Biotools 3.1 (BrukerDaltonics, Bremen, Germany).

### Proteolytic activity

In order to evaluate the proteolytic content in human plasma from healthy subjects and ATTR patients, a Pierce Fluorescent Protease Assay Kit was used, according to the manufacturer’s instructions. Briefly, the fluorescence measures were carried out with a Fluorolog-3 (Horiba Jobin Yvon) in a quartz cuvette with 0.5 cm optical path, with standard fluorescein excitation/emission filters (485/538 nm) and for the calibration trypsin was the general protease chose. Human plasma samples were diluted 100 x times in TBS (25 mM Tris, 0.15 M NaCl, pH 7.2), trypsin standards and casein solution were also prepared in this buffer. All samples and standards were incubated with the substrate at room temperature for 20 min. The estimate of protease concentration in the sample was calculated by a linear regression with the trypsin standards and then divided by the total protein amount used on the assay (µg protease/µg protein). To evaluate a putative cryptic activity of TTR we incubated plasma samples with polyclonal antibody anti-TTR. Considering a maximum TTR concentration in plasma of 0.45 g/L [27], samples were incubated with the same molar ratio of anti-TTR antibody for 1- 2h at room temperature with stirring at 300 rpm. Protein concentration was determined by the Bradford protein assay, using BSA as standard (Bio-Rad).

## Results

### Clinical and Biochemical data

All subjects are heterozygous carriers for the V30M mutation except the controls and DLT individuals that do not bear any known TTR mutation. ATTR individuals at the time of sample collection and ATTR transplanted individual at the time of transplantation showed peripheral polyneuropathy or autonomic polyneuropathy without signs of amyloid deposition [28].

All studied TTR mutation carriers or sequential liver transplanted patients were heterozygous. To validate samples for the future work the differential TTR forms in the plasma were characterized and relatively quantified as previously described (S1 Fig) [25, 26].

We separated plasma proteins by SDS-PAGE and excised TTR protein band. In the control and OLT MALDI-FTICR mass spectra, two peptides sequences of WT TTR were found, one with *m/z* 1366.759 corresponding to the sequence GSPAINVAVHVFR and a peptide with *m/z* 1494.853 corresponding to the sequence with a trypsin miss cleavage. The MALDI-FTICR mass spectra of the TTR tryptic map obtained for the ATTR and DLT samples patients with a V30M substitution presents an additional peptide, not observed in the control and OLT mass spectra at m/z 1398.732, corresponding to the 32.056 Da mass shift resulting from the V30M substitution. Hence, this peptide corresponds to the mutant peptide with the sequence GSPAINVAMHVFR, characteristic of the TTRV30M variant. The presence of a methionine residue in this peptide sequence is further confirmed by the observation of a peak with a 17.002 Da mass increment from the mutant peptide (m/z 1414.726, which corresponds to the oxidation of the methionine thioester. We also observed a third peptide (m/z 1526.826) related to the same sequence, but with a trypsin miss cleavage (GSPAINVAMHVFRK). The peptide characteristic of WT TTR (1366.759 sequence GSPAINVAVHVFR) is clearly observed in the sample of ATTR and DLT individuals, as well as the peptide characteristic of V30M TTR variant (S1 Fig) (S1 Table). This confirmed at the proteome level that all subjects are heterozygous carriers for the V30M mutation except the controls and OLT individuals that do not bear any known TTR mutation. As previously described [29], we performed a relative quantification of both TTR forms in circulation by using the relative intensity of the peak with m/z of 1366.7556 (WT TTR) to a TTR peptide present in all mass spectra acquired (such as the peak of 1394.6178 m/z).

### 2-DE plasma proteomic profile of ATTR patients

**Fig 1:**
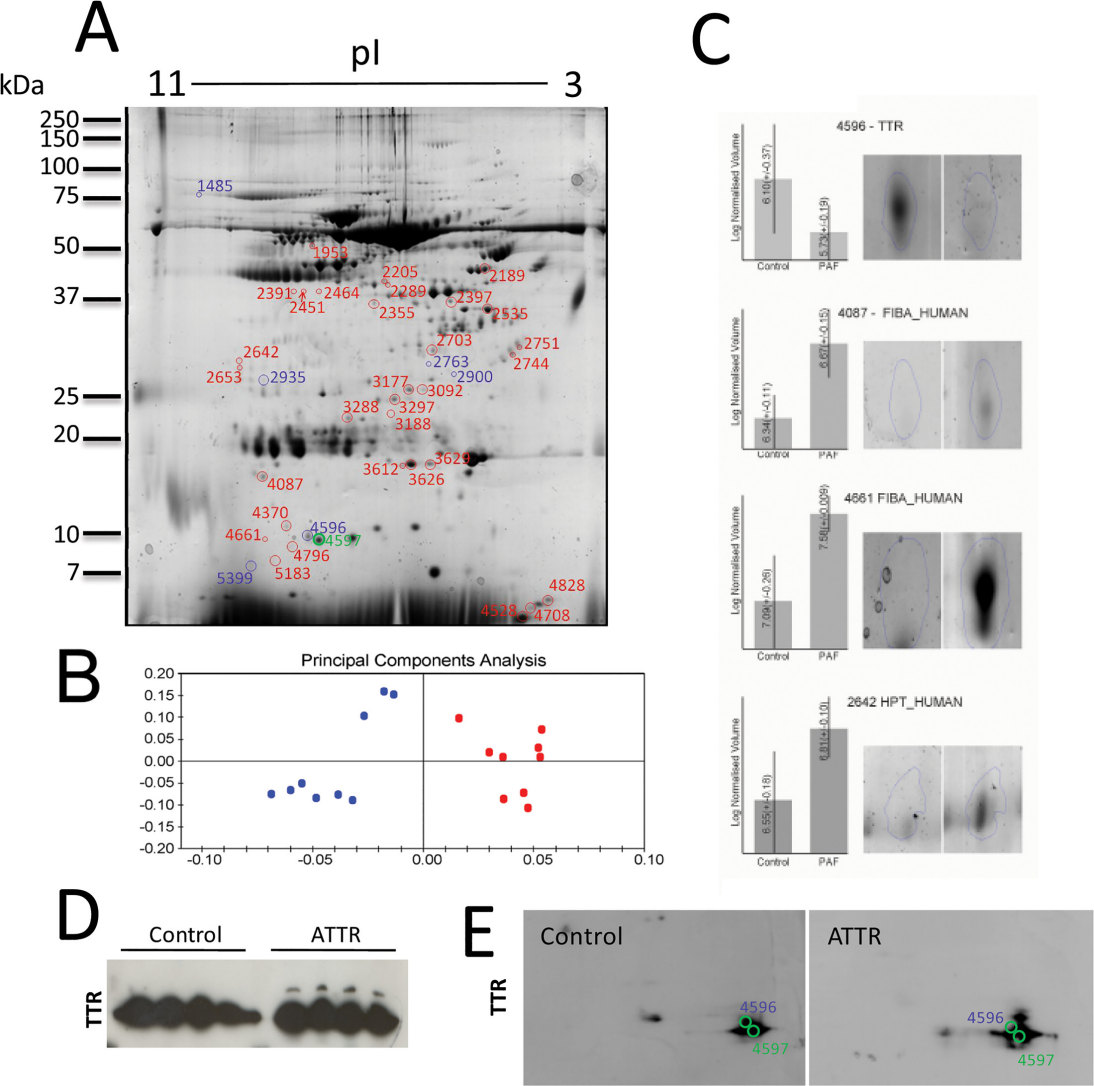
Proteome analysis of plasma from ATTR individuals. A– 2D-PAGE analysis of plasma proteins. Labeled spots show a statistically significant variation (p<0.05) and a minimal fold variation of 1.5. These spots were excised, tryptic digested and proteins identified by MS/MS analysis. Average normalized volumes and protein identifications are presented in Table 1. B–Principle component analysis (PCA) of the 2D results. Each data point in the PCA represents the global expression values for all spots with a significant ANOVA value (p<0.05). A separation between the control and the ATTR individuals is clearly observed. C- 2D image analysis of four protein spots and normalized volumes, shown as examples. D-Over expression of western blot analysis of plasma from four control and four FAP individuals to detect TTR. E—Western blot analysis of a 2DE of serum from four control and four FAP individuals to detect TTR with super imposition of spots identified as TTR in 2DE.

Plasma proteins from controls and ATTR were separated by 2-D PAGE and analyzed with the SameSpots image analysis software. Fig 1A represents a standard gel image obtained by the use of the SameSpots analysis software, combining the Gaussian images of each gel. This latter was used as a reference master map for comparison of each 2-DE gel image from ATTR and controls, in the search of differentially expressed plasma proteins. The protein spot patterns were reproducible among individuals within a study group. Principal component analysis (PCA) revealed that control individuals show a marked difference from ATTR patients. We observed clusters of 3 replicates in each group, each cluster corresponding to one individual, with the three assays corresponding to the three experimental replicates performed (Fig 1B). Similarity between 2-D PAGE gels was very high, since 95% of spots were detected in 80% of gels from individual samples. An ANOVA analysis with a cutoff at 1.5 (*p* < 0.05) revealed 42 differentially expressed spots (Table 1) between ATTR and control individuals, as shown in Fig 1C. Table 1 contains informations of the identified proteins, including their accession number, matched peptides as well as theoretical and experimental molecular weight.

**Table 1:** Differentially expressed proteins identified by MALDI-TOF-TOF MSMS.

Spot	Identification	Accession No.	T.MW (kDa)	Gel MW (kDa)	Protein Score	%seq	Pep.	Total Ion Score	Anova (p)	Fold	Average Normalized Volumes	Average Normalized Volumes	Previously identified as chaperone Ref.
											Control	ATTR	
4596	Transthyretin	TTHY_HUMAN	15,8	15	897	69	10	777	0,016	3.6	3,232e+007	8,818e+006	
2935	Haptoglobin	HPT_HUMAN	45,2	45	982	42	16	863	0,001	2,8	6,921e+006	1,964e+007	47
2535	Vitamin D-binding protein	VTDB_HUMAN	52,9	52	710	43	18	570	0,006	2,0	1,520e+006	3,095e+006	
2703	Serum albumin	ALBU_HUMAN	69.3	50	749	27	18	422	0,008	3,2	2,175e+006	6,901e+005	
1485	Serotransferrin	TRFE_HUMAN	77.0	75	155	22	15	90	0,045	1,7	7,406e+006	4,377e+006	
1485	Serum albumin	ALBU_HUMAN	69.3	75	235	25	13	161	0,045	1,7	7,406e+006	4,377e+006	
2205	Serum albumin	ALBU_HUMAN	69.3	70	961	39	27	746	0.008	2,5	1,990e+006	5,030e+006	
2900	Serum albumin	ALBU_HUMAN	69.3	65	848	32	22	709	3,068e-004	2,2	2,709e+006	5,942e+006	
2763	Serum albumin	ALBU_HUMAN	69.3	70	641	26	19	548	4,667e-005	2,4	5,163e+006	1,234e+007	
2397	Serum albumin	ALBU_HUMAN	69.3	70	1,360	49	32	1075	4,258e-005	2,3	1,187e+007	2,713e+007	
4796	Serum albumin	ALBU_HUMAN	69.3	15	665	22	16	578	0,004	3,6	1,503e+006	5,353e+006	
3177	Serum albumin	ALBU_HUMAN	69.3	40	130	11	6	104	0,015	2,1	1,077e+006	2,230e+006	
3297	Serum albumin	ALBU_HUMAN	69.3	40	817	34	24	632	3297	1,7	3,754e+006	6,367e+006	
3188	Serum albumin	ALBU_HUMAN	69.3	38	605	36	23	441	0,003	1,9	3,548e+006	6,586e+006	
3288	Serum albumin	ALBU_HUMAN	69.3	38	401	20	9	349	0,030	1,6	2,091e+006	3,425e+006	
4528	Serum albumin	ALBU_HUMAN	69.3	15	255	12	9	212	1,231e-004	3,1	2,718e+006	8,476e+006	
4708	Serum albumin	ALBU_HUMAN	69.3	15	437	18	11	409	1,420e-005	2,7	1,317e+006	3,576e+006	
4828	Serum albumin	ALBU_HUMAN	69.3	15	198	8	4	167	0,004	3,4	1,197e+007	4,074e+007	
4370	Serum albumin	ALBU_HUMAN	69.3	15	245	10	8	217	5,990e-004	2,1	1,287e+006	2,702e+006	
2355	Serum albumin	ALBU_HUMAN	69.3	70	1,090	49	29	819	5,785e-004	1,8	2,414e+006	4,411e+006	
2289	Serum albumin	ALBU_HUMAN	69.3	70	273	37	18	165	0,005	1,9	1,907e+007	3,649e+007	
2289	Fibrinogen beta chain	FIBB_HUMAN	55.9	70	649	34	17	491	0,005	1,9	1,907e+007	3,649e+007	33
2653	Serum albumin	ALBU_HUMAN	69.3	50	268	26	15	186	2,886e-004	1,8	2,465e+006	4,408e+006	
2653	Fibrinogen alpha chain	FIBA_HUMAN	95.0	50	348	15	21	280	2,886e-004	1,8	2,465e+006	4,408e+006	33
4661	Fibrinogen alpha chain	FIBA_HUMAN	95.0	15	179	10	11	150	8,835e-005	2,7	1,449e+007	3,863e+007	33
5183	Fibrinogen alpha chain	FIBA_HUMAN	95.0	12	463	13	12	424	0,001	2,6	3,175e+006	8,099e+006	33
5399	Fibrinogen alpha chain	FIBA_HUMAN	95.0	12	317	12	9	283	0,007	2,1	6,090e+005	1,304e+006	33
2189	Fibrinogen gamma chain	FIBG_HUMAN	51,5	70	1,330	38	17	1071	0,010	2,3	1,107e+007	2,510e+007	33
2642	Fibrinogen alpha chain	FIBA_HUMAN	95.0	65	429	16	15	336	0,002	1,7	3,881e+006	6,531e+006	33
2469	Fibrinogen alpha chain	FIBA_HUMAN	95.0	70	848	32	21	713	0,023	1,7	8,390e+005	1,463e+006	33
2451	Fibrinogen alpha chain	FIBA_HUMAN	95.0	70	817	25	32	645	0,008	1,7	1,316e+006	2,258e+006	33
2391	Fibrinogen alpha chain	FIBA_HUMAN	95.0	70	758	20	31	665	0,003	2,1	1,945e+006	4,119e+006	34, 47
1952	Alpha-2-macroglobulin	A2MG_HUMAN	163,3	150	1,110	47	39	913	0,017	1,8	2,855e+007	5,029e+007	33
2645	Apolipoprotein H	APOH_HUMAN	38,3	38	455	22	10	315	0,004	2,0	7,407e+006	1,444e+007	
4087	Fibrinogen alpha chain	FIBA_HUMAN	95.0	35	847	33	17	792	3,631e-005	2,2	2,261e+006	4,981e+006	33
4087	Ig gamma-2 chain C region	IGHG2_HUMAN	35,9	35	281	44	7	258	3,631e-005	2,2	2,261e+006	4,981e+006	
5144	Ig kappa chain C region	IGKC_HUMAN	11,6	10	286	67	8	218	3,338e-004	1,9	4,355e+007	8,450e+007	
3626	Ig gamma-3 chain C region	IGHG3_HUMAN	41,2	38	213	35	13	194	0,009	2,6	2,479e+007	6,468e+007	
3612	Ig gamma-2 chain C region	IGHG2_HUMAN	35,9	38	225	43	9	194	0,007	2,5	1,952e+006	4,932e+006	
3092	Complement Factor H	CFAH_HUMAN	139,0	40	469	19	33	388	0,008	1,8	6,147e+006	1,101e+007	
2751	Clusterin	CLUS_HUMAN	52,4	50	193	14	8	168	0,006	3,6	1,302e+006	4,738e+006	36,41,46,57
2744	Clusterin	CLUS_HUMAN	52,4	50	1,050	42	32	896	5,749e-005	3,3	1,729e+006	5,649e+006	36,41,46,57
3249	Inter-alpha-trypsin inhibitor heavy chain	ITIH4_HUMAN	103,3	40	718	46	12	654	0,028	1,7	4,555e+006	2,755e+006	
3190	Protein AMBP	AMBP_HUMAN	39.0	40	432	29	8	374	7,341e-004	1,8	2,759e+006	4,961e+006	
2642	Alpha-1-antitrypsin	A1AT_HUMAN	46,7	50	150	32	18	87	0,002	1,7	3,881e+006	6,531e+006	37

We observed that the protein spot presenting the higher variation between the two groups (3.6 fold–spot 4596) was WT TTR, in agreement with our previous observation that wild type TTR corresponds to around 60% of the pool of circulating TTR in ATTR individuals [21]. Both TTR variants were identified in spot 4597 in ATTR individuals, and only WT TTR was identified in this spot in control individuals, however this spot volume did not change between the two groups. Moreover, TTR presents a higher heterogeneity in ATTR patients since several PTMs were described for mutant, [30, 31].

Western blot analysis, from SDS-PAGE and 2DE that revealed that TTR bands in 1D electrophoresis and spots in 2D electrophoresis are more heterogeneous in ATTR individuals (Fig 1D and 1E).

The presence of N-glycosylated forms of TTR, as described in the literature, can certainly contribute for the heterogeneity in the 2DE, since the proteins glycoforms are usually present as 'trains' of spots in the first dimension, differing also in their molecular weight [32].

Several proteins, previously described as extracellular chaperones overrepresented in ATTR plasma. In recent years, a substantial body of evidence leads to the suggestion that certain acute-phase components including fibrinogen [33], alpha 2 macroglobulin (AAG) [34] and haptoglobin [35] possess chaperone action being able to inhibit in a dose-dependent manner stress-induced aggregation of a number of unrelated target proteins. Clusterin, an ubiquitous extracellular mammalian—but not an acute phase protein—also displays a well-characterized chaperone function [36]. It was also described that alpha 1 anti-trypsin (AAT), which we also observed overrepresented, might exhibit chaperone like activity, preventing aggregation of Aβ peptides and related amyloidogenic proteins [37].

The vitamin D binding protein was overrepresented in the plasma of ATTR individuals and that alpha-trypsin inhibitor heavy chain was under expressed. The similar result was recently obtained for the differential proteome of serum of Alzheimer individuals [38].

**Fig 2:**
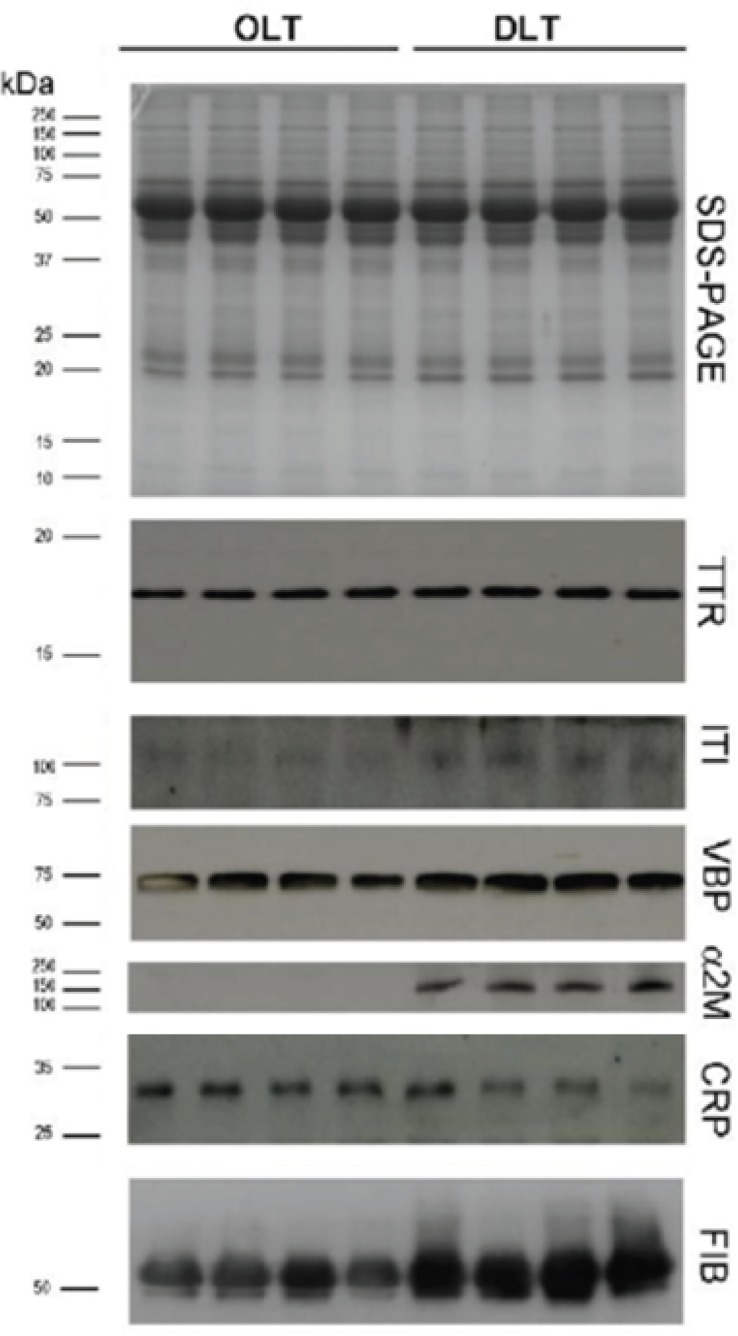
Extracellular Chaperones are overrepresented in DLT individuals—SDS-PAGE analysis of plasma proteins from four individuals after orthotic liver transplantation (OLT) and four individuals after domino transplantation (DLT) and Western blot to detect TTR, Fibrinogen, Alpha 1 anti-trypsin (ITI), Vitamin D binding protein (VBP), alpha 2 microglobolin and C-reactive protein (CRP). Molecular weight markers are represented on the left hand of the figure and included in the western blots as blue markers.

We previously observed that fibrinogen expression is increased in ATTR patients [39] and this result was confirmed in this work, in which several spots of fibrinogen were over expressed in ATTR patients. Since most TTR is produced by the liver, the progression of the disease can be halted by OLT, which leads to the disappearance of the V30M TTR form of the transplant recipient [40]. To obviate the shortage of livers available for transplantation, DLT was introduced in which a liver from a ATTR patient is transplanted to a patient with liver failure. DLT introduces mutated TTR variants in circulation, increasing the risk of ATTR development. To evaluate if DLT individuals presented the same increased levels of the proteins observed in ATTR individuals we collected samples of individuals that were subjected to OLT and DLT and monitored expression of fibrinogen, Vitamin D binding protein, Alpha 2 macroglobolin and alpha 1 anti-trypsin by western blot analysis. As shown in Fig 2, these proteins are also found to be overrepresented in DLT individuals, but not in OLT individuals. TTR and the C-reactive protein, a prototypic acute phase protein, levels do not present any significative difference between DLT and OLT individuals.

Altogether our observations strength the hypothesis that extracellular chaperones are overrepresented to cope with misfolded protein clearance, as previously suggested in Alzheimer disease [41] and ATTR [39].

### ATTR individuals present higher plasma proteolytic activity

**Fig 3:**
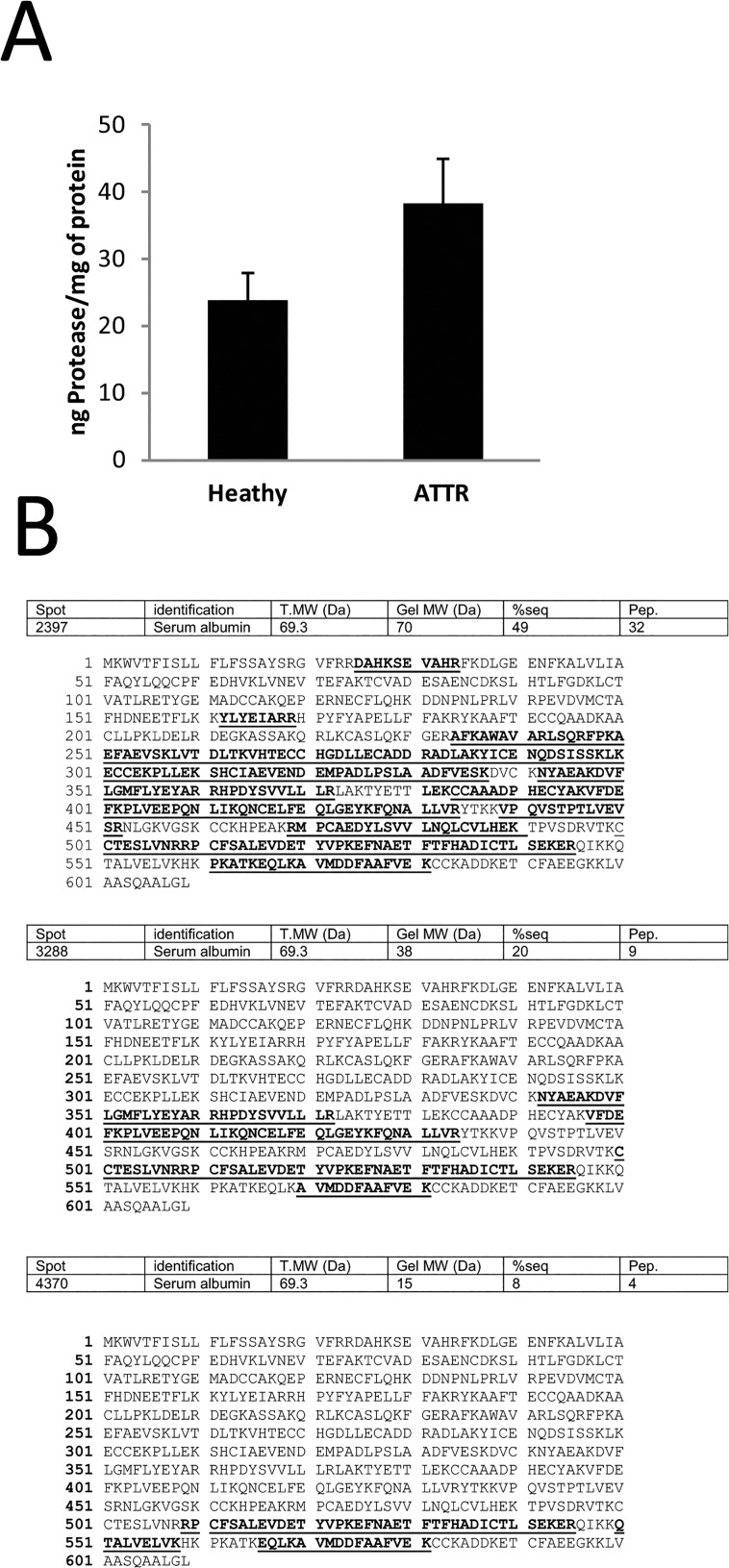
Plasma from ATTR individuals presents a higher proteolytic activity. A- Proteolytic activity of control and ATTR plasma measured by fluorescent protease assay kit. B–Sequence coverage obtained for three spots identified as albumin with decreased molecular weight.

A striking observation in 2DE of plasma from ATTR relatively to controls is the presence of a large set of proteins with a lower apparent molecular weight than it’s predicted value, which could indicate the presence of proteolytic fragments. For the majority of these cases we observed a decrease in sequence coverage for proteins identified by mass spectrometry and these proteins were identified at a lower molecular weight than the theoretical one (Fig 3B). We can observe the same protein in several spots with decreasing molecular mass. Take for example serum albumin which was detected in the spots 2205, 3177 and 4796 with approximate molecular weight of 70 kDa, 40 kDa and 15 kDa, respectively. Also fibrinogen alpha chain was detected in several spots of decreasing mass (spots 2391, 2653 and 4461 with respective molecular masses of 70, 50 and 15 kDa). These observations made us question about a higher proteolytic activity in the plasma of ATTR individuals. We tested this hypothesis by measuring the proteolysis of casein in our plasma samples and, as shown in Fig 2C, we observed a significant increase in the proteolytic activity in the plasma of ATTR patients with a p-value for the t-test of 9.2583E^-12^.

TTR has also been established as a cryptic protease, and since one of the proteins found differentially represented in the plasma of ATTR patients was APO-AI known to be its substrate [42] we evaluated the possibility of this increase in the proteolytic activity in ATTR plasma due to a differential TTR cryptic protease activity. We measured the proteolytic activity in the plasma in the presence of anti TTR polyclonal antibody. No differences were observed in the proteolytic content in the plasma of both control and ATTR individuals (data not shown). Proteasomes have also been detected in normal human blood plasma and designated circulating proteasomes; these have a comparatively low specific activity, a distinct pattern of subtypes and their exact origin is still enigmatic [43].

Incubation of plasma with MG132, to evaluate if the proteolysis observed was due to a higher activity of the circulating proteasome, also presented no differences in the proteolytic content in the plasma of both control and ATTR individuals (data not shown).

## Discussion

The major problem associated with the 2-DE analysis of plasma is the presence of abundant proteins, such as albumin and the wide dynamic range in abundance of other proteins as well as the tremendous heterogeneity of its predominant glycoproteins. Abundant proteins in the sample may interfere with the detection of less abundant proteins of interest and for this reason preclearance methods to deplete the most abundant plasma proteins have become widespread [44]. However, it has been reported that albumin removal is associated also with the removal of some other proteins that might be important as well [45]. For this reason, in this work, we chose not to remove any protein prior to 2-DE analysis. In our case we evaluate the plasma proteome of ATTR patients relatively to healthy individuals. ATTR patients used in this study are all heterozygous for TTR mutation (V30M). We previously reported that ATTR individual’s present around 40% less WT TTR relatively to control individuals [26, 29]. In fact we observed the spot that presents the higher variation between the two groups (3.6 fold) was identified as TTR, validating the experiment.

Several extracellular chaperones, as clusterin, haptoglobin and alpha2M were found to be overrepresented. All these extracellular chaperones have the ability to bind misfolded proteins and thereby inhibit inappropriate protein-protein interactions, preventing aggregation and maintaining proteins in solution. They demonstrate the ability to influence amyloid formation *in vitro* [46,47] and are found colocalized with clinical amyloid deposits *in vivo*. The amyloid condition associated to ATTR results from the accumulation of a protein prone to form amyloid fibers, and so chaperones and other elements of proteostasis may be overwhelmed by the larger amount of non native protein present in the extracellular space. We may speculate that these chaperones are overrepresented in ATTR plasma to overcome the larger amount of a protein prone to form amyloid fibers, and act collectively. Some of these chaperones were found overrepresented in several other amyloid diseases, as clusterin in Alzheimer [48, 49]. This hypothesis is supported by the overexpression of Vitamin D Binding Protein and the under expression of IHRP as was found in the plasma of Alzheimer´s patients. Altogether these data point to sticking similarities between amyloid diseases.

Similarly to the extracellular molecular chaperone HP, AMG and clusterin, AAT and other serpin proteins have also been found to be associated with pathological protein deposits in various diseases [50–52]. It has been proposed that such deposits represent the failed attempts of molecular chaperones to maintain the solubility of misfolded proteins and peptides under disease specific conditions of high molar substrate excess [46, 53]. Previous reports have indicated that AAT can inhibit the formation of amyloid fibrils [54]. AAG and AAT has also been identified as the aggregation inhibitor of the amyloidogenic human alpha-atrial natriuretic peptide [55]. In another study, albumin, AAT, and immunoglobulins at physiological plasma concentrations showed to be potent inhibitors of amyloid beta-peptide (Ab) polymerization [56]. Curiously, we also observed albumin and several Iggs differentially expressed and these extracellular chaperones have been described as overrepresented in several conformational diseases [57]. It was suggested that this overexpression counteracted the presence of a protein prone to form amyloid diseases.

DLT using grafts from ATTR patients was first performed in 1995 [58] and more than 400 domino transplant procedures had been carried out by the end of 2005, according to the Familial Amyloidotic Polyneuropathy World Transplant Registry. The age of onset in ATTR Portuguese patients, where TTR shows a substitution of methionine by valine at position 30, ranges from the late 20s to early 40s. It was, therefore, expected that liver grafts explanted from ATTR patients would function in recipients without formation of amyloid fibrils for a long period. However, several recent reports [59, 60] indicate that amyloid deposition or ATTR symptoms appear in domino recipients much sooner than in ATTR individuals. Our observation that some extracellular chaperones are overrepresented in individuals that were transplanted with a sequential liver, starting from that point on to produce mutated TTR makes it highly plausible that the increased levels of these chaperones is a response to additional request of extracellular chaperone activities under these pathological conditions [39]. Human fibrinogen specifically interacts with and suppresses aggregation of a wide spectrum of stressed proteins [61]. It was proposed that human fibrinogen can interact with prefibrillar species during the fibril formation process, redirecting the aggregation process. It is also interesting to note that high levels of human fibrinogen are also related to both Alzheimer’s disease, vascular dementia [62] and ATTR [39]. The same can be speculated to all the others extracellular chaperones observed to be overrepresented, as clusterin, that was previously implicated in fibrillogenesis and extracellular misfolded protein clearance in Alzheimer disease [63] and its overexpression was described to have possible protective role in transthyretin deposition in ATTR [57], although significantly lower levels of circulating clusterin were associated with amyloid deposition in the heart in ATTR, is what appears to reflect a unique difference in amyloidosis pathology, dependent on organ involvement [64]. In SSA, amyloid fibers present a high percentage of ATTR fragments starting at position 46, 49 and 52, with the presence of a widely variable proportion of full length TTR [65]. There is still some controversy in knowing if the proteolytic cleavage occurs before or after the fibers formation, but according to our results is seems likely that it occurs already in the bloodstream, before fiber formation [66].

We believe that the overexpression of extracellular chaperone is a hallmark of conformational diseases and that in the future the most effective therapies for these diseases will be based on preventive approaches rather than downstream solutions. Moreover these common features hold great potential to be used as biomarkers for amyloid diseases.

## Supporting Information

S1 Fig Relative quantification of TTR variants n the plasma. A–Schematic representation of the methodology. B- Peptide mass fingerprint of the TTR protein band after tryptic digest from plasma. The ion 1366.759 is present in control, ATTR, OLT and DLT individuals. This corresponds to the peptide with the sequence GSPAINVAVHVFR. The ion 1398.732 is present only in ATTR and DLT individuals. This ion corresponds to the peptide with the sequence GSPAINVAMHVFR.
(EPS)

S2 Fig Original uncropped and unadjusted blots. (EPS)

S1 Table Sequence coverage for TTR in the plasma of Control, ATTR individuals orthotropic liver transplantation (OLT) individuals and domino liver transplantation (DLT) individuals. Unicode sp|P02766| without 20 amino acids of the signal peptide.
(DOCX)
