# Supplementary material for: Transthyretin Amyloidosis: Chaperone Concentration Changes and Increased Proteolysis in the Pathway to Disease
Source: PLoS One. 2015 Jul 6;10(7):e0125392. doi: 10.1371/journal.pone.0125392 (PMC4492746; doi:10.1371/journal.pone.0125392)
Supplement: S1 Table — Unicode sp|P02766| without 20 amino acids of the signal peptide. (DOCX) [file pone.0125392.s003.docx]

Supplementary Table 1-Sequence coverage for TTR in the plasma of Control, ATTR individuals orthotropic liver transplantation (OLT) individuals and domino liver transplantation (DLT) individuals. Unicode sp|P02766|without 20 amino acids of the signal peptide.

|  | Sequence | Range | Control | ATTR | OLT | DLT |
| --- | --- | --- | --- | --- | --- | --- |
| 1366.76 | GSPAINVAVHVFR | 22–34 | X | X | X | X |
| 1394.62 | AADDTWEPFASGK | 36–48 | X | X | X | X |
| 1398.73 | GSPAINVAMHVFR | 22–34 |  | X |  | X |
| 1414.73 | GSPAINVAMHVFR – oxid M | 22–34 |  | X |  | X |
| 1494.85 | GSPAINVAVHVFRK | 22-35 | X | X | X | X |
| 1522.72 | KAADDTWEPFASGK | 26–39 | X | X | X | X |
| 1526.83 | GSPAINVAMHVFRK | 22–35 |  | X |  | X |
| 2451.21 | ALGISPFHEHAVVFTANDSGPR | 81-103 | X | X | X | X |
| 2455.15 | TSESGELHGLTTEEEFVEGI YK | 49-70 | x | X | x | X |
